# Increasing doctors working in specific rural regions through selection from and training in the same region: national evidence from Australia

**DOI:** 10.1186/s12960-021-00678-w

**Published:** 2021-10-29

**Authors:** Matthew R. McGrail, Belinda G. O’Sullivan

**Affiliations:** 1 The University of Queensland Rural Clinical School, 78 on Canning St, Rockhampton, QLD 4700 Australia; 2 The University of Queensland, Rural Clinical School, Locked Bag 9009, Toowoomba, QLD DC 4350 Australia

**Keywords:** Rural workforce, Medical education, Selection, Training, Distribution, Grow your own workforce, Social accountability, Rural origin, Doctors

## Abstract

**Background:**

‘Grow your own’ strategies are considered important for developing rural workforce capacity. They involve selecting health students from specific rural regions and training them for extended periods in the same regions, to improve local retention. However, most research about these strategies is limited to single institution studies that lack granularity as to whether the specific regions of origin, training and work are related. This national study aims to explore whether doctors working in specific rural regions also entered medicine from that region and/or trained in the same region, compared with those without these connections to the region. A secondary aim is to explore these associations with duration of rural training.

**Methods:**

Utilising a cross-sectional survey of Australian doctors in 2017 (*n* = 6627), *rural region of work* was defined as the doctor’s main work location geocoded to one of 42 rural regions. This was matched to both (1) *Rural region of undergraduate training *(< 12 weeks, 3–12 months, > 1 university year) and (2) *Rural region of childhood origin *(6+ years)*,* to test association with returning to work in communities of the same rural region.

**Results:**

Multinomial logistic regression, which adjusted for specialty, career stage and gender, showed those with > 1 year (RRR 5.2, 4.0–6.9) and 3–12 month rural training (RRR 1.4, 1.1–1.9) were more likely to work in the same rural region compared with < 12 week rural training. Those selected from a specific region and having > 1-year rural training there related to 17.4 times increased chance of working in the same rural region compared with < 12 week rural training and metropolitan origin.

**Conclusion:**

This study provides the first national-scale empirical evidence supporting that ‘grow your own’ may be a key workforce capacity building strategy. It supports underserviced rural areas selecting and training more doctors, which may be preferable over policies that select from or train doctors in ‘any’ rural location. Longer training in the same region enhances these outcomes. Reorienting medical training to selecting and training in specific rural regions where doctors are needed is likely to be an efficient means to correcting healthcare access inequalities.

## Background

‘Grow your own’ strategies in health workforce development have emerged globally as a critical solution for communities to build the capacity of their local rural health workforce. They began in rural medicine in 1990 [1–7], which involved selecting health students from specific rural regions and training them in the same regions for extended periods, to improve local retention. This was considered more efficient and productive than trying to recruit doctors with no connection to rural medicine and the specific region, to work across the broader scope of skills needed in rural communities [8–11]. Equally, ‘growing your own’ was considered preferable over relying on compulsory rural practice terms which can cause a mismatch of skills, lower satisfaction and poorer retention of doctors [12, 13].

The World Health Organisation (WHO) endorses ‘grow your own’ type strategies for rural and remote areas, including in its updated global recommendations applicable to all countries [14, 15]. In 2018, it also sponsored the development of a checklist to foster implementation of ‘grow your own’ training, in low and middle income countries (LMICs) [7]. This checklist has been informally tested successfully in high-income countries, but not validated to date. However, the research underpinning the WHO recommendations does not evaluate the relationship between specific regions of rural origin, training and work. Instead, most evidence uses ‘any’ rural location as their measure of success, whereby success can be achieved by doctors working in the rural regions with greatest amenity. It is critical to explore whether origin and training from specific rural regions improves doctor supply to those regions, so as to establish the specific value of rural training strategies to local or similar communities. Building the quality of the evidence about this is also necessary to advocate decentralised medical training when metropolitan policymakers and universities may wish to hold resources and power for medical training in large cities. Decentralising training resources maintains those resources in rural areas for the ongoing benefit of those communities, which additionally has potential to lead to better investment in health care in those rural communities [16].

‘Grow your own’ aligns with the movement to increase ‘socially accountable’ education, where clinical education and training systems are structured to deliver on social (community), rather than institutional objectives. The WHO first defined the concept of social accountability in 1995 [17, 18], and socially accountable medical training programs have since globally expanded [10, 19]. A suite of tools to support formative evaluation of socially accountable programs (not restricted to medicine) has been developed since 2008 by the Training for Health Equity network (THEnet) [8, 20–24]. Social accountability strategies align with increased rural recruitment and retention [25]; however, the strength of evidence of research evaluating outcomes of these strategies remains relatively weak. To date it is limited to mainly single-institution studies of early-career graduates with limited adjustment for confounding factors, but with a few exceptions, is done without connecting specific regions of selection and training to their region of work [26–28]. More national-scale, cross institutional and controlled research is essential to underpin higher quality evidence and more widespread adoption of the strategy.

Japan is an example of one country that has had a long-term national policy of quota selections from specific regions (with some conscripted return of service to the same region); however, evidence of their outcomes is not specific to whether doctors work in the same region [29]. The only other research about the value of ‘grow your own’ was a single-institution study of junior doctors from Monash University, Australia. It was the first study to show a statistical association between sub-regions of selection and training and working in the same region [30]. Notably, training in a sub-region for longer (2 years versus 1 year) increased rates of returning to work in the same rural region. In addition, those with both training and selection connections to a region were substantially more likely to return working there than those with just a training connection [31, 32]. Research done at national scale, across the whole medical workforce (working at all career stages), would increase confidence to policy-makers considering implementing ‘grow your own’ policies. In addition, it is important that such research recognises the different capacity to remain in a rural region between those in general practice (GP) or other specialties [33].

With this background in mind, this national study aims to explore whether doctors working in specific rural regions also entered medicine from that region and/or trained in that region, compared with those without these connections to the region. A secondary aim is to explore these associations with duration of rural training.

## Methods

This study uses data from a large national longitudinal survey of doctors, established in 2008 to inform policy and planning, called Medicine in Australia: Balancing Employment and Life (MABEL). In 2008, the whole Australian medical workforce was invited to participate in this survey, and 10 498 (19.4% response rate) responded, a highly representative cohort [34]. After 2008. The annual survey maintained a 70–80% participant retention through to its completion in 2018, with some annual top-up (including from new graduates). MABEL was approved by the University of Melbourne Faculty of Business and Economics Human Ethics Advisory Group (ref. 0709559) and the Monash University Standing Committee on Ethics in Research Involving Humans (ref. CF07/1102-2007000291).

The current research uses the data from the 2017 MABEL survey (*n* = 9512), which was the first to ask participants about rural training: ‘*Did you participate in rural placements as part of your basic medical degree?*’ Respondents were able to list up to three locations (town, state, postcode) and to detail how long they had spent in each one (< 12 weeks; 3–12 months; > 1 university year). The survey already collected information about rural origin and a range of relevant covariates. This study included all clinically active doctors who had graduated from an Australian university (excluding overseas-trained doctors), representing doctors spanning all career stages, specialties, practice settings and locations.

### Context of this study

**Fig. 1:**
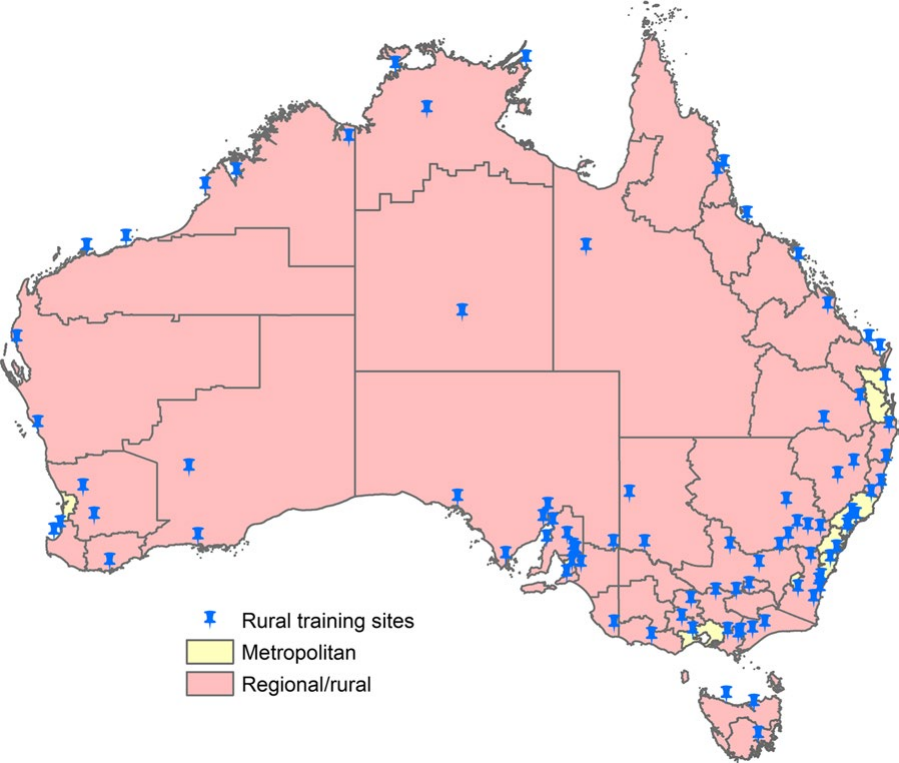
Map of study’s 54 regions (42 rural) and their alignment with rural training sites. Footnote: Rural training occurs in many additional locations to those noted, mostly in primary care, such as via the John Flynn placement program (since 1997) and small group training now overseen by the Rural Clinical Schools (since about 2000)

Australia provides a useful case study for this research. It has had a relatively stable national rural medical training policy since 2000, which the government contracts to most medical schools nationally to provide training in specific rural regions through a decentralised Rural Clinical School program [35]. It also had a national medical workforce survey (MABEL) which provides the essential data variables to investigate this research question [32]. The national rural training policy requires medical schools to enroll ≥ 25% rural background students (although it does not specify they must be from any specific rural region) and provide at least 12 month rural training to ≥ 25% of each cohort. This training occurs within each medical school’s defined geographic region, typically including both a training hub in a larger regional centre and smaller training sites in rural and remote areas (Fig. 1). In addition, since 1997 Australian medical students have had opportunities for annual (repeated) rural or remote training experiences via the John Flynn Placement Program [36]. Prior to these policies, less commonly Australian doctors could train rurally through localised opportunities or of their own accord, which were mostly of shorter duration (< 3 months) [32, 37].

### Defining regions and training locations

All Australian locations were allocated to one of 54 regions (Fig. 1). First, the six major cities (Adelaide, Brisbane, Canberra, Melbourne, Perth and Sydney), with boundaries defined by the Australian Bureau of Statistics (ABS), were coded as ‘metropolitan’ regions. Second, consistent with national rurality scales, six other large population centres nearby (< 2 h’ drive) to major cities (Sunshine Coast, Gold Coast, Newcastle, Central Coast, Wollongong and Geelong) were also defined as ‘metropolitan’. All other locations were coded to one of 42 ‘rural’ regions, with region boundaries aligning strongly with jurisdictional hospital and health service regions and regional specialist care networks in Australia (Box 1, functional areas specific to healthcare) [38]. They also moderately aligned with 88 hubs of undergraduate rural training under Australia’s national rural training policy (Fig. 1).

Box 1: Defining rural regions The boundaries for Australia’s 42 rural regions were defined using a combination of the Australian Bureau of Statistics’ (ABS) Australian Statistical Geography Standard (ASGS) SA3 and SA4 borders:

- SA3s represent approximately 30–130 000 residents, designed to closely match local functional areas and local government area boundaries.
- SA4s represent 100–300 000 residents, designed to define regional labour markets and are wholly an aggregate of SA3 sub-regions.
- Most regions were defined wholly by a single SA4 boundary (*n* = 38)
- Where an SA4 region was too large (*n* = 4), SA3 boundaries were used to split it into two regions

### Defining return to rural region

*Rural region of work* was defined by geocoding the doctor’s main location of work (town/suburb and postcode, < 1% missing data) and where this corresponded to one of the 42 rural regions.

*Rural region of training* was determined based on up to three rural locations doctors recounted. Each location, and the corresponding duration doctors trained there was geocoded to a rural region.

*Return to rural region* was counted if the doctor’s rural region of work matched with any of three rural regions of training they reported. Different rural region indicated a doctor was working rurally, but had not trained there. Where a match occurred, the longest duration of training in that region was recorded. If unmatched, longest rural duration was recorded. The three possible outcomes from matching were return to: (1) same rural region; (2) different rural region and; (3) metropolitan.

*Rural region of origin* was determined by doctors reporting “*the main rural area where you lived up until school leaving age*” (town/suburb and postcode), categorised ‘yes’ only if the doctor noted at least 6 rural childhood years and then geocoded to a rural region. Six years was chosen, consistent with evidence that this is the minimum duration associated with doctors working rurally [39]. To test sensitivity, total rural childhood years was additionally analysed with categories 1–5 years, 6–11 years and 12–18 years.

As per for training, *return to rural region* was counted if the doctor’s rural region of work matched the rural region of origin.

Finally, rural region of training and rural region of origin were tested together. *Return to rural region* was counted if the doctor’s rural region of work matched either their rural region of origin or one of their rural regions of training.

### Other key covariates

Work location often relates to specialty and career stage [40], so covariates included: pre-registrar (working in hospitals, no fixed specialty), GP (either registrar, or independent practice); and non-GP specialties (either registrar, or independent practice). In Australia, pre-registrar doctors are mostly able to nominate, where they work, including choosing most regions but may prefer metropolitan locations to increase opportunities for entry to non-GP specialty training [41, 42]. Differences by gender were additionally explored.

### Analyses

All analyses used Stata SE 15.1 for Windows (Stata Corp, College Station, Texas) and *α* = 0.05 for statistical significance. Descriptive statistics were used to present basic rates of doctor’s rural region of work and rural region of training or rural region of origin. Multinomial logistic regression models (by necessity, limited to participants undertaking rural training), measured associations with returning to work in the same rural region, a different rural region or metropolitan region, adjusting for key covariates.

## Results

There were 6627 usable responses, after excluding respondents who were overseas-trained (21.2%), non-clinically active (extra 10.7%) and invalid work location (extra 1.0%). Table 1 shows respondent characteristics and rates of working rurally which were highest in general practice (37.0%) and among doctors who either trained rurally > 1 year (43.7%) or had a childhood rural origin (39.2%).

**Table 1:** Summary of included participant characteristics

	Participants (*n*, %)	Working rural (*n*, %)
Career group		
Pre-registrar	776 (12%)	176 (22.7%)
Registrar	1130 (17%)	222 (19.7%)
Independent practice	4721 (71%)	1163(24.6%)
Specialty		
General practice	2288 (35%)	847 (37.0%)
Non-GP specialty	3563 (54%)	538 (15.1%)
N/A (pre-registrar)	776 (12%)	176 (22.7%)
Gender		
Male	3244 (49%)	773 (23.8%)
Female	3383 (51%)	788 (23.3%)
Longest rural region of training during medical school		
Nil	2523 (38%)	469 (18.6%)
< 12 weeks	2329 (35%)	546 (23.4%)
3–12 months	1180 (18%)	286 (24.2%)
> 1 year	595 (9%)	260 (43.7%)
Rural region of origin (childhood 6+ years)		
Yes	1380 (21%)	541 (39.2%)
No	4805 (72%)	906 (18.9%)
Missing (unknown)	442 (7%)	114 (25.8%)
Region of work (*n* = 54)		
Metropolitan	5066 (76%)	N/a
Rural	1561 (24%)	N/a

Table 2 shows that amongst respondents working in rural regions, many had either the same rural region of training or same rural region of origin. First, 388/1558 (25%) with a rural region of work had trained in the same rural region, and second, 177/567 (31%) with both a rural region of work and rural origin had the same region of origin (6+ years). There was a consistent dose–response effect, with increased duration of training in a specific rural region (< 12 weeks, 3–12 months, > 1 year) associated with increased rates of working in the same rural region (6.2%, 8.3%, 24.4%). Similarly, increased years in a rural region of origin (1–5, 6–11, 12–18 years) was associated with increased rates of working in the same rural region (5.9%, 7.0%, 12.4%). All rates of working in the same rural region were higher when training duration both increased and was combined with rural origin, reaching 28.2% for those trained > 1 year rurally and of rural origin.

**Table 2:** Crude associations between rural region of training or rural region of origin and rural region of work

	Same rural region of work	Different rural region of work	Metropolitan region of work
Duration of training in rural region			
Observed	388	1170	5069
Nil (metropolitan only)	N/A^#^	467 (18.5%)	2056 (81.5%)
< 12 weeks	145 (6.2%)	400 (17.2%)	1784 (76.6%)
3–12 months	98 (8.3%)	188 (15.9%)	894 (75.8%)
> 1 year	145 (24.4%)	115 (19.3%)	335 (56.3%)
Rural region of origin (childhood years)			
Observed	177	1381	5069
Nil rural child years	N/A^a^	801 (18.2%)	3598 (81.8%)
1–5	24 (5.9%)	76 (18.8%)	304 (75.3%)
6–11	24 (7.0%)	101 (29.5%)	218 (63.6%)
12–18	129 (12.4%)	289 (27.8%)	621 (59.8%)
Unknown region	N/A	114 (25.8%)	328 (74.2%)
Combined rural region of training and rural region of origin			
Observed	461	1097	5069
Nil and metro origin	N/A^a^	305 (16.2%)	1581 (83.8%)
< 12 weeks and metro origin	75 (4.3%)	273 (15.5%)	1415 (80.3%)
3–12 months and metro origin	50 (5.5%)	119 (13.1%)	738 (81.4%)
> 1 year and metro origin	77 (17.6%)	58 (13.2%)	303 (69.2%)
Nil and rural origin	33 (6.8%)	113 (23.1%)	343 (70.1%)
< 12 weeks and rural origin	87 (16.2%)	111 (20.6%)	340 (63.2%)
3–12 months and rural origin	60 (18.4%)	62 (19.0%)	204 (62.6%)
> 1 year and rural origin	79 (28.2%)	56 (20.0%)	145 (51.8%)

^a^N/A = where observing returning to work in the same region is impossible, either because they undertook no rural training or they also were classified as having a metropolitan origin

Table 3 shows the multivariate model of factors associated with working in the same rural region. After adjusting for specialty, career stage and sex, the relative risk ratio (RRR) of working in the same rural region of training was RRR 1.42 (1.08–1.88) for 3–12 month training and RRR 5.22 (95% CI 3.95–6.89) for > 1 year in the same region, compared with < 12 weeks. In contrast, only longer duration (> 1 year rural training) was associated with working in a different rural region. Rural region of origin was also independently associated with an increased rate of working in the same rural region (RRR 3.24, 95% CI 2.54–4.12). GPs (both registrars training to become GPs and qualified GPs) were more likely to be working in the same rural region as their medical school training than pre-registrars. In contrast, non-GPs (both registrars and qualified) were less likely to be working in the same rural region of training. Females were also less likely to work in the same rural region of origin/training than males.

**Table 3:** Multivariate multinomial logistic regression model of return to same rural region of work as rural region of training experienced during basic medical training

Ref: work in metropolitan	Same rural region of work (RRR, 95% CI)	Different rural region of work (RRR, 95% CI)
Career stage (ref = Pre-registrar)		
Non-GP: registrar	0.24 (0.14–0.39)***	0.76 (0.53–1.09)
Non-GP: independent	0.63 (0.44–0.90)*	1.08 (0.80–1.46)
GP: registrar	2.70 (1.67–4.38)***	4.37 (2.87–6.64)***
GP: independent	2.09 (1.51–2.89)***	3.53 (2.64–4.72)***
Female (ref = Male)	0.75 (0.59–0.94)*	0.79 (0.66–0.94)**
Rural region of training (ref = < 12 weeks)		
3–12 month training	1.42 (1.08–1.88)*	0.99 (0.81–1.21)
> 1 year training	5.22 (3.95–6.89)***	1.54 (1.19–1.98)**
Rural region of origin (ref = no)^a^	3.24 (2.54–4.12)***	2.32 (1.91–2.81)***

*N* = 4097 (Excluded: nil rural training); RRR: relative risk ratio; *p < 0.05, **p < 0.01, ***p < 0.001

^a^Analysis was repeated with rural origin separated into 6–11 years and 12–18 years; however, this revealed similar effect sizes of same rural region of work: 6–11 years (RRR 3.16, 2.08–4.81)***, 12–18 years (RRR 3.26, 2.51–4.24)***

Table 4 shows multivariate results of combining rural region of training (durations) with rural region of origin. At each increment (< 12 weeks, 3–12 months and > 1 year) of training in a rural region and having a rural origin, were 5.5, 7.6 and 17.4 times more likely (RRR) to work in the same rural region as either of these connection points, compared to those with < 12 week rural training time and metropolitan origin.

**Table 4:** Multivariate multinomial logistic regression model of return to same rural region work as rural region of training experienced during basic medical training and rural region of origin (6 years+ in childhood)

Ref: metropolitan region of work	Same rural region of work (RRR, 95% CI)	Different rural region of work (RRR, 95% CI)
Career stage (ref = Pre-registrar)		
Non-GP: registrar	0.24 (0.15–0.39)***	0.81 (0.56–1.18)
Non-GP: independent	0.63 (0.45–0.89)**	1.13 (0.82–1.55)
GP: registrar	2.34 (1.45–3.77)***	4.91 (3.21–7.51)***
GP: independent	2.14 (1.56–2.93)***	3.76 (2.78–5.08)***
Female (ref = Male)	0.76 (0.61–0.96)*	0.78 (0.65–0.93)**
Rural training (< 12 weeks and metro region of origin)^a^		
3–12 months and metro origin	1.46 (1.02–2.08)*	0.97 (0.77–1.22)
> 1 year and metro origin	6.95 (4.93–9.78)***	1.52 (1.12–2.08)**
< 12 weeks and rural origin	5.52 (3.92–7.77)***	1.84 (1.38–2.44)***
3–12 months and rural origin	7.60 (5.11–11.3)***	1.96 (1.36–2.82)***
> 1 year and rural origin	17.4 (11.6–26.1)***	2.70 (1.78–4.11)***

*N* = 4104 (Excluded: nil rural training); RRR: relative risk ratio; *p < 0.05, **p < 0.01, ***p < 0.001

^a^Analysis was repeated with rural region of origin separated into 6–11 years and 12–18 years; however, this revealed similar effect sizes of same rural region of work: for example, > 1 year and 6–11 years (RRR 17.7)***, > 1 year and 12–18 years (RRR 17.0)***

## Discussion

This is the first national study about the potential gains to regions that invest in ‘grow your own’ medical workforce strategies. This study uses control groups and national regional definition to isolate the strong net value of ‘grow your own’ strategies for building the capacity of doctors in specific rural regions. This type of evaluation differs greatly to most other research in this field, which have reported on graduates working in ‘any rural’ location, mostly done at a single-institution level, with limited controls contributing to the current ‘weak’ rating of evidence in WHO guidelines [15]. These new findings provide unique evidence to support global policy recommendations, specific to medicine. They support related evidence that long-term placements in communities can strongly shape their medical identity, particularly in rural locations, whereby doctors acculturate and build relevant skills and networks, as well as setting up families, all of which support retention [32, 43, 44].

This research adds that ‘grow your own’ value is strengthened by increasing the duration of training periods in the region, where the community may need more doctors. This is also the case for those with increased years of origin in that same region*.* These findings may support underserved communities to advocate for medical programs that select and extend the period of training in their specific region, for efficient and targeted outcomes. Within Australia, there currently is growth of ‘end-to-end’ medical training, such as the new Murray Darling Medical Schools Network [45]. Our results are encouraging that targeted selection and training in specific regions through such strategies are likely to substantially increase the retention of graduates in those regions.

The findings can be applied to many countries globally, as they provide objective evidence to guide countries starting to implement and refine their plans for rural medical education. They build on emerging evidence from other similar national (Japan) and province (Northern Ontario) level strategies that also show promising returns to their rural regions [28, 29]. In Australia, which has established national rural training policies over 20 years, the findings usefully suggest that efficiencies could be gained by requiring its medical schools to increasingly target selecting rural background students from the same regions, where they provide rural training (rather than any rural region of origin). Some programs are leading the way, such as the new Northern Territory (NT) Medical Program which prioritises first NT indigenous applicants and second NT non-indigenous applicants [2]. Other wholly regional medical programs also have processes to select high proportions of rural origin students from their jurisdictions, and show encouraging retention at the macro regional level [46–49].

The scale of this study removes concerns of institutional or jurisdictional bias, which is common in other literature on this topic. Whilst corroborating with the author’s previous single-institution and early-career research about ‘grow your own’ [30], the current research is strengthened using national data across the whole medical workforce. This smoothed the effects across all different cohorts, adjusting for effect moderators, such as career stage, specialty and gender, with appropriate comparison groups enabling greater generalisability for policymaking. The results concur with the global push for increasing investment in distributed medical education and regional medical programs. Apart from being an efficient mechanism to overcome inequalities in health service access, decentralising training infrastructure may provide other benefits, such as expanding job opportunities in locations, where work is most difficult to find. Local investment of training resources will have both a direct and indirect economic benefit within such regions and is preferable to outreach models [50]. This has particular relevance for lifting social and economic productivity of rural communities, as well as improving health in LMICs [7].

Perhaps unsurprisingly, GPs were the major contributor to those observed working in the same region as previously trained or lived in their childhood. Moreover, GPs sustained similar rates of working in the same region, regardless of career stage. This may relate to the fact that GP training in Australia is organised within geographic boundaries, with 50% of trainees needing to choose rural regions [51]. This provides opportunities for these doctors to train/work in the same rural region for longer. In contrast, non-GP registrars were much less likely to be working in the same region and qualified non-GP specialists were still below the rates of pre-registrar doctors. Training in non-GP specialties involves more limited rural opportunities and greater dependence on city-based infrastructure and staffing for practice [52, 53]. Strengthened regional training opportunities for this group is likely to enhance ‘grow your own’ outcomes.

This study’s results come from a high-income country (HIC) with a longstanding (20 years) national rural training policy across government-supported medical schools. Validation of these is required in other HICs and LMICs to test whether comparable rates of working in the same rural region are seen; however, this requires substantial data infrastructure which is often lacking. It is possible for HICs to partner with LMICs to mentor and support relevant research infrastructure, such as occurs via THEnet and the Medical Education Partnership Initiative with the USA [21, 54]. Although implementing processes to select doctors from rural regions may be a relatively cheap intervention, the enrollment cost for rural students (particularly in private medical schools) may deter uptake of medical training, especially if decentralised training options near their community do not exist. Implementing high quality rural training requires skilled rural supervisors, clinical infrastructure, funding, positive working conditions and engaged communities [7, 55]. Any costs of developing rural pathways to ‘grow your own’ should be advocated by presenting the projected health, social and economic returns for participating communities [50].

Limitations within this study include that it only measured undergraduate rural training and childhood rural origin as the primary rural connections. No other regional linkages (such as high school graduation, partner’s childhood location, or postgraduate training location) were available, providing conservative estimates. Further research could test whether more contact points within rural regions will produce stronger results. Whilst these data are drawn from a longitudinal project design, rural training questions were only added in 2017 and thus this research was limited to a cross-sectional analysis and could not measure long-term retention (sustainability). Respondents reported up to three rural training periods categorically, supporting collection of consistent data through a national survey, but this limited the capacity to test the sensitivity of different training periods across different programs. The study only included doctors and further research could be done to understand this phenomenon in relation to other health workers. This study offers multiple design strengths, but primarily are its national design with regions and broad participation across all career stages and specialties. Further research is recommended using a qualitative approach to explore in more detail why a ‘grow your own’ outcome is not always achieved and thus what strategies can strengthen such outcomes.

## Conclusions

This study provides the first national-scale empirical evidence suggesting that ‘grow your own’ strategies are associated with increased medical graduates being retained in the specific regions of rural origin and training, even after adjusting for specialty, career stage and gender. It supports the expectation that selecting doctors from and training them in specific rural regions, are likely to be efficient and effective strategies to grow the medical workforce in regions that need more doctors. Longer duration of rural training in the same region and more years of rural origin in that region are also likely to enhance doctors’ propensity to work in the same region. This strengthens the case for countries to reorient medical training to selecting and training in specific rural regions, where doctors are needed, as a means of correcting healthcare access inequalities.

## Data Availability

The data sets used and/or analysed during the current study are available from the corresponding author on reasonable request.

## Abbreviations

ABS: Australian Bureau of Statistics
ASGS: Australian Statistical Geography Standard
GP: General Practitioner
HIC: High Income Country
LMIC: Low and Middle Income Country
MABEL: Medicine in Australia: Balancing Employment and Life (study)
NT: Northern Territory
RRR: Relative Risk Ratio
THEnet: Training for Health Equity network
WHO: World Health Organization
